# A 50-kb deletion disrupting the *RSPO2* gene is associated with tetradysmelia in Holstein Friesian cattle

**DOI:** 10.1186/s12711-020-00586-y

**Published:** 2020-11-11

**Authors:** Doreen Becker, Rosemarie Weikard, Christoph Schulze, Peter Wohlsein, Christa Kühn

**Affiliations:** 1 Leibniz Institute for Farm Animal Biology (FBN), Institute of Genome Biology, Wilhelm-Stahl-Allee 2, 18196 Dummerstorf, Germany; 2 Department of Pathology, University of Veterinary Medicine, Hannover, Germany; 3 Present Address: Landeslabor Berlin-Brandenburg, Frankfurt (Oder), Germany; 4 Rostock, Faculty of Agricultural and Environmental Sciences, Rostock, Germany

## Abstract

**Background:**

Tetradysmelia is a rare genetic disorder that is characterized by an extremely severe reduction of all limb parts distal of the scapula and pelvic girdle. We studied a Holstein Friesian backcross family with 24 offspring, among which six calves displayed autosomal recessive tetradysmelia. In order to identify the genetic basis of the disorder, we genotyped three affected calves, five dams and nine unaffected siblings using a Bovine Illumina 50 k BeadChip and sequenced the whole genome of the sire.

**Results:**

Pathological examination of four tetradysmelia cases revealed a uniform and severe dysmelia of all limbs. Applying a homozygosity mapping approach, we identified a homozygous region of 10.54 Mb on chromosome 14 (*Bos taurus* BTA14). Only calves that were diagnosed with tetradysmelia shared a distinct homozygous haplotype for this region. We sequenced the whole genome of the cases’ sire and searched for heterozygous single nucleotide polymorphisms (SNPs) and small variants on BTA14 that were uniquely present in the sire and absent from 3102 control whole-genome sequences of the 1000 Bull Genomes Project, but none were identified in the 10.54-Mb candidate region on BTA14. Therefore, we subsequently performed a more comprehensive analysis by also considering structural variants and detected a 50-kb deletion in the targeted chromosomal region that was in the heterozygous state in the cases’ sire. Using PCR, we confirmed that this detected deletion segregated perfectly within the family with tetradysmelia. The deletion spanned three exons of the bovine *R-spondin 2* (*RSPO2*) gene, which encode three domains of the respective protein. R-spondin 2 is a secreted ligand of leucine-rich repeats containing G protein-coupled receptors that enhance Wnt signalling and is involved in a broad range of developmental processes during embryogenesis.

**Conclusions:**

We identified a 50-kb deletion on BTA14 that disrupts the coding sequence of the *RSPO2* gene and is associated with bovine tetradysmelia. To our knowledge, this is the first reported candidate causal mutation for tetradysmelia in a large animal model. Since signalling pathways involved in limb development are conserved across species, the observed inherited defect may serve as a model to further elucidate fundamental pathways of limb development.

## Background

Dysmelia is a congenital disorder that results in deformities of the limbs and occurs sporadically in various species, but the cause of this disorder is often unclear. These limb malformations are characterized by the loss of anatomic structures and functions. Different forms of dysmelia are distinguished depending on the number of affected limbs and the part of the limb that is malformed or missing [1]. Various limb malformation phenotypes ranging from deficiencies of the upper and lower limb have been described in humans and various animal species (Table 1) [2–10].

**Table 1:** Inherited limb deficiencies in various species

Disorder	Species	Mode of inheritance	Phenotype	Gene	References
Amputated	Cattle	AR	Absence of limbs distal from elbow and hock joints	NK	https://omia.org/OMIA000036/9913/; [36, 39, 40]
Acroteriasis congenita	Cattle	AR	Absence of the distal parts of the limbs, head deformities	NK	https://omia.org/OMIA000010/9913/; [37, 40]
Ectromelia	Cattle	NK	Congenital absence of distal parts of the limbs, cleft lip/palate, mandibular hypoplasia, scoliosis	NK	https://omia.org/OMIA001126/9913/; [38, 40]
	Dog	NK	Front legs very small or absent	NK	https://omia.org/OMIA001126/9615/; [7]
Hemimelia	Dog	NK	Congenital absence of all or part of the distal part of a limb	NK	https://omia.org/OMIA000450/9615/; [6]
	Goat	NK	Congenital absence of all or part of the distal part of a limb	NK	https://omia.org/OMIA000450/9925/; [3]
	Sheep	NK	Congenital absence of all or part of the distal part of a limb	NK	https://omia.org/OMIA000450/9940/; [9, 63]
Limbless	Chicken	AR	Absence of limbs, shortened upper beak	NK	https://omia.org/OMIA000602/9031/; [13]
Peromelia	Cattle	NK	Absence of the distal parts of the limbs	NK	https://omia.org/OMIA000786/9913/; [2]
	Goat	AR	Absence of the distal parts of the limbs	NK	https://omia.org/OMIA000786/9925/; [5]
Footless	Mouse	AR	Abnormal limb morphology, abnormal kidney development, cleft palate, absence of all nails	*Rspo2*^*ftls*^	https://informatics.jax.org/allele/key/29601; [10]
*Fgf-10*-deficiency	Mouse	AR	Complete absence of limbs, abnormal lung development	*Fgf10*^*tm1Wss*^	https://informatics.jax.org/allele/key/3008;
Renal dysplasia-limb defects syndrome	Human	AR	Growth retardation, complete phocomelia of upper limbs, renal dysplasia, abnormal genitalia	NK	https://omim.org/entry/266910; [48]
Posterior amelia	Human	AR	Absence of hindlimbs, hypoplastic or absent pelvic bones, hypoplasia of the sacrum, lung hypoplasia	*TBX4*	https://omim.org/entry/601360; [50]
Tetraamelia syndrome 1	Human	AR	Limb agenesis, cleft lip/palate, diaphragmatic defect, lung, renal and adrenal agenesis, pelvic hypoplasia, urogenital defects	*WNT3*	https://omim.org/entry/273395; [20, 47]
Tetraamelia syndrome	Human	AR	Rudimentary appendages or complete absence of the limbs, bilateral agenesis of the lungs, cleft lip/palate, ankyloglossia, mandibular hypoplasia, microretrognathia, labioscrotal fold aplasia	*RSPO2*	https://omim.org/entry/618021; [22, 49]
Al-Awadi/Raas-Rothschild syndrome	Human	AR	Severe malformations of upper and lower limbs with severely hypoplastic pelvis and abnormal genitalia	*WNT7A*	https://omim.org/entry/276820; [21]

Disorders have been selected from the Online Mendelian Inheritance in Animals catalogue (OMIA), Online Mendelian Inheritance in Man (OMIM) records and Mouse Genome Informatics database, respectively; *AR* autosomal recessive, *NK* not known

Tetradysmelia, which is characterized by a severe reduction of all limb parts distal of the scapula and the pelvic girdle, is an extremely rare limb malformation in cattle [11]. Similar defects have been described only in humans and chickens (Table 1) [12, 13]. The genetic regulation of limb development is conserved across species [14] and governed by a three-dimensional signalling system that defines proximodistal, anteroposterior and dorsoventral axes. Outgrowth is promoted by a fibroblast growth factor (FGF) signal allowing proximal/distal patterning [15, 16]. Expression of the *sonic hedgehog* (*Shh*) gene produces a polarizing signal and controls anterior/posterior patterning [17, 18], whereas Wnt signalling controls dorsal/ventral patterning [19]. Genetic studies in humans and in model organisms have demonstrated that disruption of many genes related to and involved in this complex limb signalling system can lead to marked alterations in limb size and shape as well as complete failure of the limb development (Table 1) [20–23]. To date, candidate gene analyses have not identified the genetic cause of tetradysmelia in cattle [24–26].

The objective of our study was to elucidate the genetic background of Holstein Friesian calves affected with tetradysmelia by using a homozygosity mapping approach in combination with whole-genome sequencing (WGS) in order to identify the genetic causal variant and contribute to knowledge about limb development.

## Methods

### Animals

**Fig. 1:**
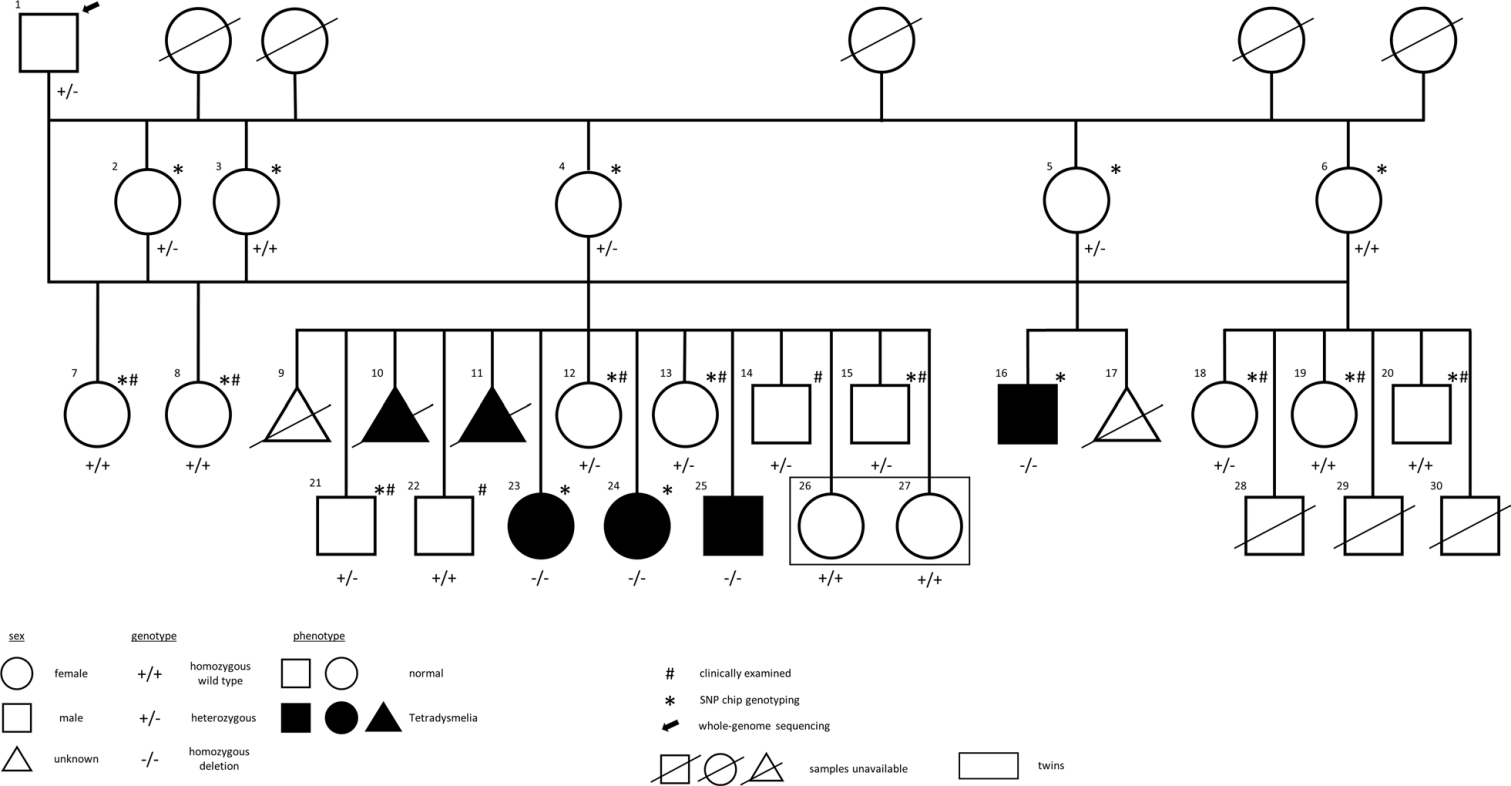
Pedigree of the Holstein Friesian backcross family. Family tree of the Holstein Friesian backcross family (animal no. 1 to 30). Females are shown in circles, males in squares, unknown sexes in triangles. Filled symbols represent affected animals. Symbols with a diagonal line show animals, for which no DNA sample was available. Animals indicated with a hashtag (#) were clinically examined. Necropsy was performed on animal no. 16, 23, 24 and 25. Animals indicated with an asterisk (*) were used for SNP genotyping. All animals are related to a single founder male, for which the whole genome was sequenced (black arrow). Parents of affected calves showed a normal phenotype. Genotypes for the 50-kb deletion confirmed by PCR are displayed under each symbol for animals with an available DNA sample

The studied animals belong to a Holstein Friesian backcross family born in the 1990s with 24 offspring, among which six stillborn calves lacked all four limbs. The male founder, a proven sire from a private artificial insemination station, was mated to five of its daughters (Fig. 1). After their second or third parturition at the age of 5 to 6 years, the daughters were superovulated according to a conventional embryo transfer (ET) scheme and inseminated with their sire’s sperm. The resulting embryos were recovered at day 7 and checked morphologically. After storage at -196 °C, embryos with a normal shape and development were transferred to 15–21 months old recipients that were kept on four farms. Pregnancies were confirmed by rectal palpation.

### Clinical investigation and necropsy

Of the 24 calves born in the backcross family, 18 did not display limb deformations and 11 of these were available for clinical investigation (shown with a # in Fig. 1). Four stillborn calves that lacked limbs were investigated by necropsy (animal number 16, 23, 24 and 25 in Fig. 1) after photographs and radiographs were taken. For two of the six stillborn calves, necropsy was not performed and the only available description of the malformation was that provided by the farmer. Sex was not recorded on farms for four of the calves (triangles in Fig. 1).

### DNA extraction

Blood samples were collected using standard clinical venepuncture techniques from the sire, the five dams and from 11 unaffected offspring. In addition, we collected tissue samples (ear notches) from six offspring (unaffected n = 2; tetradysmelia n = 4) and obtained a sperm sample from the sire of the backcross family. Genomic DNA was isolated by phenol–chloroform standard methods [27]. DNA integrity was assessed by agarose gel electrophoresis and DNA quality and concentration were measured with a NanoDrop spectrophotometer (www.nanodrop.com). Samples with a 260/280 ratio between 1.8 and 2 were diluted to a final concentration of 50 ng/µL and were used for genotyping on a single nucleotide polymorphism (SNP) chip and PCR amplification.

### SNP genotyping and homozygosity mapping

Due to quality issues with one DNA sample from an affected calf (that was born mummified), only three tetradysmelia cases were genotyped (Fig. 1) on an Illumina BovineSNP50 v2 DNA Analysis BeadChip according to the manufacturer’s instructions (Illumina, Inc., CA, USA). In addition, we genotyped five dams and nine unaffected siblings. DNA of the sire was genotyped on the Illumina HD 777 k chip and used for whole-genome sequencing (see below). Prior to analysis of the SNP data, standard quality control settings were used to remove SNPs with a call rate lower than 90% and a minor allele frequency (MAF) lower than 1%.

A homozygosity mapping approach was applied in order to identify extended intervals of homozygosity with shared alleles. Homozygosity analysis was performed using the PLINK v1.09 software [28] on all malformed cases using the commands “--cow”, “--homozyg” and “--homozyg-group” on the dataset. Only runs of homozygosity that contained at least 30 SNPs or with a total length longer than 100 kilobases (kb) were considered. Since the Illumina BovineSNP50 v2 BeadChip annotation data are based on the UMD_3.1.1 genome reference [29] and mapping of the whole-genome sequencing data (see below) was carried out against the ARS-UCD1.2 reference genome [30], the NCBI “remap” function (https://www.ncbi.nlm.nih.gov/genome/tools/remap/) was used to convert the location of the homozygous regions to the new reference genome (ARS-UCD1.2). All subsequently reported genome positions correspond to ARS-UCD1.2.

### Whole-genome sequencing and variant calling

We sequenced the whole genome of the cases’ sire using next-generation sequencing technology. To this end, sequencing libraries were prepared at ATLAS Biolabs (https://www.atlas-biolabs.com) using the Illumina’s TruSeq DNA library preparation kit. Libraries were sequenced with a 2 × 100-bp paired end protocol on an Illumina HiSeq2000 (ATLAS Biolabs) and an Illumina HiSeq2500 (in-house). Mapping and variant calling were performed according to the 1000 Bulls Genome pipeline (https://www.1000bullgenomes.com/), i.e. 1000 bulls GATK fastq to GVCF guidelines (GATKv3.8). Briefly, reads were trimmed and filtered using the Trimmomatic 0.38 program (https://www.usadellab.org/cms/?page=trimmomatic) and then mapped to the ARS-UCD1.2_Btau5.0.1Y genome using the Burrows-Wheeler Aligner (BWA) version 0.7.17 (https://github.com/lh3/bwa). The ARS-UCD1.2_Btau5.0.1Y genome includes the Y chromosome assembly from Baylor College [31]. Samtools 1.8 (https://www.htslib.org/download/) was used to sort the mapped reads by sequence coordinates. PCR duplicates were labelled using the Picard v2.18.2 software (https://broadinstitute.github.io/picard/). Base quality recalibration (BQSR) was performed with the Genome Analysis Tool Kit (GATK version v3.8-1-0-gf15c1c3ef). Finally, SNPs and indels were called using the GATK ‘HaplotypeCaller’. Variant data were obtained in variant call format as raw calls for all samples, and sites were flagged using the variant filtration module of GATK. In addition, the average read coverage across the whole genome was calculated using the GATK DepthOfCoverage tool.

### Variant filtering

Under the assumption that the sire was a carrier of the causal variant, first we filtered the whole genome data for SNPs and small indels in 28 candidate genes that were selected based on reports of associations with limb malformations in other species and their biological function in skeletal development and formation [see Additional file 1: Table S1]. In addition, we filtered for heterozygous variants that occurred exclusively in the sire’s genome against 3102 control genomes of Run 7 of the 1000 Bull Genomes Project using bcftools (version 1.6). The Delly2 program [32] was used to detect structural variants in the BAM file of the sire’s genome sequencing reads. The search for deletions, inversions, duplications and translocations was conducted simultaneously. Structural variant analysis focused on the candidate region that was identified by homozygosity mapping. In order to validate the detected structural variants and exclude false positives, the structural variants present in the candidate region of 14 unrelated cattle genomes (n = 5 Holsteins, n = 5 Charolais, n = 4 Holstein $\times$ Charolais crosses), which were sequenced during other projects of our group, were also analysed. The Integrative Genomics Viewer (IGV) software (version 2.5.3; https://software.broadinstitute.org/software/igv/) was used to visualize potential structural variants. Furthermore, we looked for already existing structural variants in the Cow Structural Variant database (Ensembl; www.ensembl.org). Sequence read coverage of BTA14 was calculated using samtools (version 1.9).

### PCR and Sanger sequencing

Presence of the identified structural variant in the *RSPO2* gene was investigated in all available tetradysmelia family samples using PCR. PCR primers were designed using Primer3 [33] after masking repetitive regions in the target region with RepeatMasker (https://www.repeatmasker.org/).

Two primer pairs were designed. Each pair had the same common forward primer (BTA14_Del_F; 5′-TCCCTGAGCCAGTGAATTCC-3′; 5′-start: BTA14 g.56,450,454), and a different reverse primer (BTA14_DelWt_R; 5′-GTGTCCGACTCTGTGTGACC-3′; 5′-start: BTA14 g.56,451,423, and BTA14_DelMt_R; 5′-GCATCAGCGCTAAGAACTGC-3′; 5′-start: BTA14 g.56,501,362). The forward primer was placed upstream of the deletion, one reverse primer was located in the deleted region (BTA14_DelMt_R), whereas the other reverse primer (BTA14_DelWt_R) was designed to match the sequence downstream of the deletion. PCR products were amplified from genomic DNA of the sire, five dams, four cases and 13 unaffected siblings using GoTaq polymerase (Promega). The resulting PCR products were analysed on a 2% agarose gel with 0.5 µg/mL ethidium bromide. In addition, DNA samples of 182 unrelated Holstein Friesians from Mecklenburg-Western Pomerania in Germany were genotyped for the deletion.

To verify the exact boundaries of the deletion, PCR products of two homozygous cases and two heterozygous carriers were sequenced by Sanger sequencing on an ABI 3500 Genetic Analyzer capillary sequencer (Life Technologies) using the forward and reverse PCR primers as indicated above. Sequence data were analysed using the Sequencher 5.0 tool (GeneCodes).

### Statistical analysis

The mean read depths of the deleted region and its surrounding regions were calculated using the R software (version 3.6.0). T-tests were performed to determine whether the mean coverage between the deletion and the upstream sequence (BTA14: 56,350,000–56,451,028 bp), between the deletion and the downstream sequence (BTA14: 56,501,202–56,550,000 bp) and between the upstream and downstream sequence differed significantly. To visualize the results, we used the Ggplot2 (version 3.2.0) R software package.

## Results

### Backcross family

Backcrossing of a proven Holstein Friesian bull to five of its daughters via embryo transfer resulted in 24 offspring (male = 10, female = 10, unknown sex = 4) (Fig. 1). Among the 18 calves born without limb malformation, four were stillborn: one foetus was aborted at day 218 (no. 17, Fig. 1), one was born prematurely at day 250 (no. 9, Fig. 1), and a pair of twins was born at day 270 (no. 26 and 27, Fig. 1). Five stillborn calves (female = 2, male = 1, unknown sex = 2) and one aborted foetus (male = 1) displayed abnormal limb development. The occurrence of dysmelia has not been reported for the ancestors or other offspring of the sire and for the dams before the backcrossing. Based on the ratio between offspring with and offspring without limb malformations, on the fact that both sexes were affected and that the dams and sire had no malformations, we can conclude that the mode of transmission of tetradysmelia agrees with the hypothesis of an autosomal recessive inheritance pattern.

### Clinical and pathological examination

Of the 14 live-born calves (all without limb defects, see above), 11 calves (female = 6, male = 5; indicated with a hashtag (#) in Fig. 1) were available for further clinical examination. None of them showed any sign of other malformations or malfunctions. Their average birth weight was 39.7 kg and they were delivered after an average gestation period of 276.1 days.

**Fig. 2:**
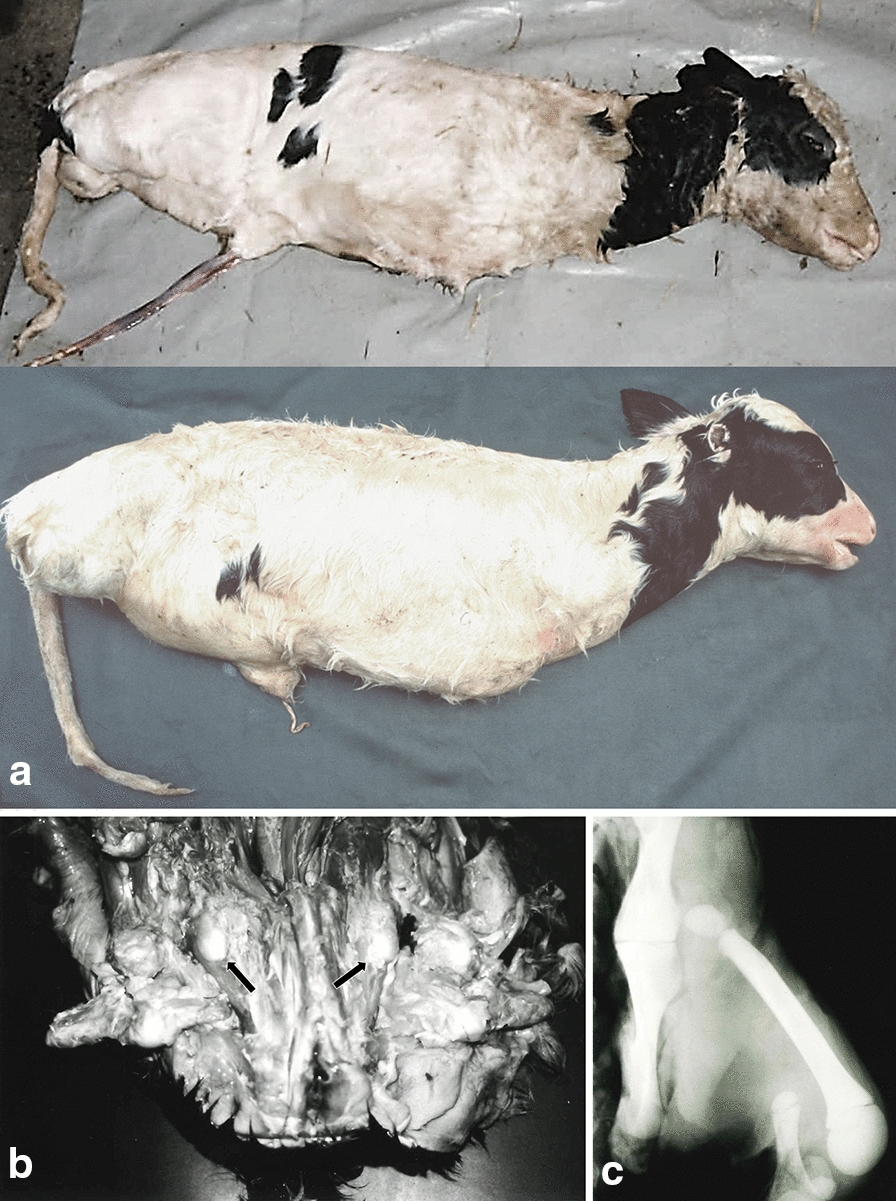
Phenotype of affected calves. **a** Stillborn calf no. 23 (top) and 24 (bottom) after 272 and 269 days of gestation, respectively, with the characteristic tetradysmelia phenotype. **b** Pelvis of bovine calf no. 16 after 270 days of gestation. No acetabulae are developed, and instead of hindlimbs only irregular nodular clumps of mixed cartilaginous and osseous tissues are present in the disorganized musculature (black arrows). **c** Radiography of left hind leg of bovine calf no. 23 after 272 days of gestation. Bones of reduced size and deformed shape are developed

Four individuals available for inspection (stillborn = 3, aborted foetus = 1; female = 2, male = 2) had obvious limb malformations (Fig. 2). In addition, farmers reported two other stillborn calves that completely lacked all four limbs (no. 10 and 11 in Fig. 1). Necropsy was performed on three stillborn calves and one aborted foetus with limb malformations (no. 16, 23, 24 and 25 in Fig. 1). Foetus no. 25 (in Fig. 1) was born mummified after 162 days of gestation with a weight of 360 g and a crown rump length of 26 cm. The other three animals (no. 16, 23, 24 in Fig. 1) were born after 269 to 272 days of gestation with a weight ranging from 21.1 to 22.6 kg. The forelimbs of animals numbered 16, 24 and 25 consisted of the scapulae only with complete lack of the distal parts of the forelimbs (Fig. 2a). Pelvic acetabulae were missing but isolated round cartilage-covered bone structures with a diameter of 3 to 4 cm were present in the disorganized musculature ventrolateral of the pelvis (Fig. 2b). Limb development had progressed further in animal no. 23, with the presence of not only both scapulae, but also the humerus of the left forelimb. Furthermore, differentiated bones (femur, tibia) of reduced size and shape were present in both hindlimbs (Fig. 2c). In addition, animal no. 23 lacked the symphyseal fusion of the rostral ends of the mandibulae and animal no. 25 showed a mild brachygnathia superior.

### Mapping of the tetradysmelia locus

Genotyping of all individuals recorded as offspring of the sire (no. 1 in Fig. 1), which were available for SNP genotyping and were used for mapping the defect (Fig. 1), confirmed the parentage as indicated in the pedigree records. A chromosomal region that harboured the causal mutation and its flanking chromosomal segments was expected to be identical-by-descent (IBD) in all affected animals, because these were all offspring of the same sire, who also sired the dams, and segregation analysis had indicated that the disorder was inherited in an autosomal recessive mode (see above). Therefore, a homozygosity mapping approach was applied to determine the position of the mutation in the bovine genome. In total, 54,609 SNPs were genotyped in three malformed individuals, five dams and nine unaffected siblings. After quality control, 45,132 SNPs remained for analysis. We searched for regions of homozygosity with simultaneous allele sharing on the autosomes in the three cases and identified 16 genomic regions. The size of the homozygous blocks ranged from 85 kb to 10.54 Mb. Six of the homozygous regions could be excluded because the cases were in fact homozygous, but not for the same alleles. To further exclude homozygous blocks, the identified regions in the dams of the three cases and the unaffected siblings were also examined. Dams are expected to be heterozygous, whereas the unaffected siblings could be either heterozygous or homozygous for alleles that differ from those carried by cases. Only one identified region fulfilled these criteria: on BTA14, all malformed individuals shared identical homozygous genotypes for 179 SNPs corresponding to a 10.54-Mb interval ranging from 52.42 to 62.96 Mb and containing 68 loci.

### Whole-genome sequencing

Due to the large size of the region associated with tetradysmelia and the lack of obvious candidate genes in this region, whole-genome sequencing (WGS) of the sire was carried out to detect variants within the candidate interval on BTA14. WGS enabled us to investigate further genomic regions, if the primary search of a plausible causal variant in the initial candidate interval on BTA14 failed. We received 879 million paired-end reads corresponding to a mean coverage of 29.66x across the genome. Following sequencing and mapping, heterozygous SNPs as well as heterozygous small indel variants detected in the sire were filtered against 3102 control genomes from the 1000 Bull Genomes project. Subsequently, we identified 1153 heterozygous variants that were exclusively present in the genome of the sire. Of those 1153 variants, 28 were located on BTA14 but no SNP or small indel variant was found in the candidate region or was located in plausible candidate genes across the genome.

### Identification of structural variants

**Fig. 3:**
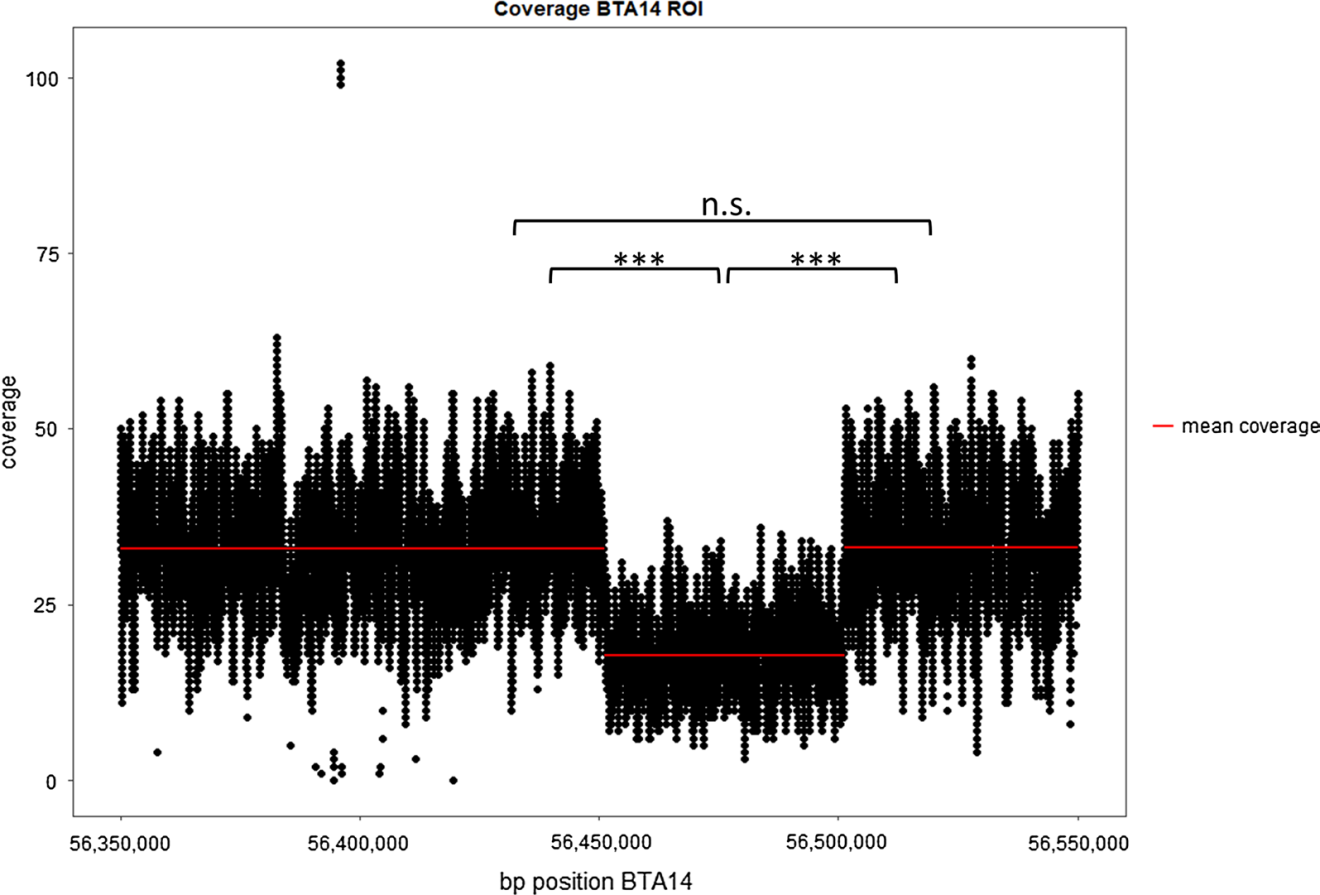
Coverage plot for bp positions 56,350,000 bp to 56,550,000 bp on BTA14 of the sire with offspring affected by tetradysmelia. Data were derived from whole-genome sequencing. The sequences include the region of the deletion and flanking sequences upstream and downstream. Red horizontal lines indicate mean coverage. Mean coverage decreases at 56.45 Mb and increases at 56.50 Mb significantly (***p < 0.001); n. s. not significant

As our initial analysis pipeline did not detect any plausible causal variant in the candidate genome region or plausible candidate gene, we used the Delly2 package to search for heterozygous structural variants. In the sire, 42 deletions, three inversions and two duplications were found in the critical 10.54-Mb region on BTA14. Analysis of the candidate region in 14 unrelated cattle genomes, the search for structural variants already reported in the Cow Structural Variant database (www.ensembl.org), and visual inspection of the candidate region allowed us to further reduce the number of candidate structural variants. Only one structural variant, a heterozygous deletion of about 50 kb between 56.45 and 56.50 Mb on BTA14, remained after filtering [see Additional file 2: Fig: S1]. The read coverage of the deleted region was compared with the upstream (56.35–56.45 Mb) and downstream (56.50–56.55 Mb) sequences. At 56.45 Mb, the mean coverage drops significantly (p < 0.001) from 32.98x to 17.79x, and at 56.50 Mb it increases significantly (p < 0.001) to an average of 33.03x (Fig. 3).

### Characterization of the structural variant

**Fig. 4:**
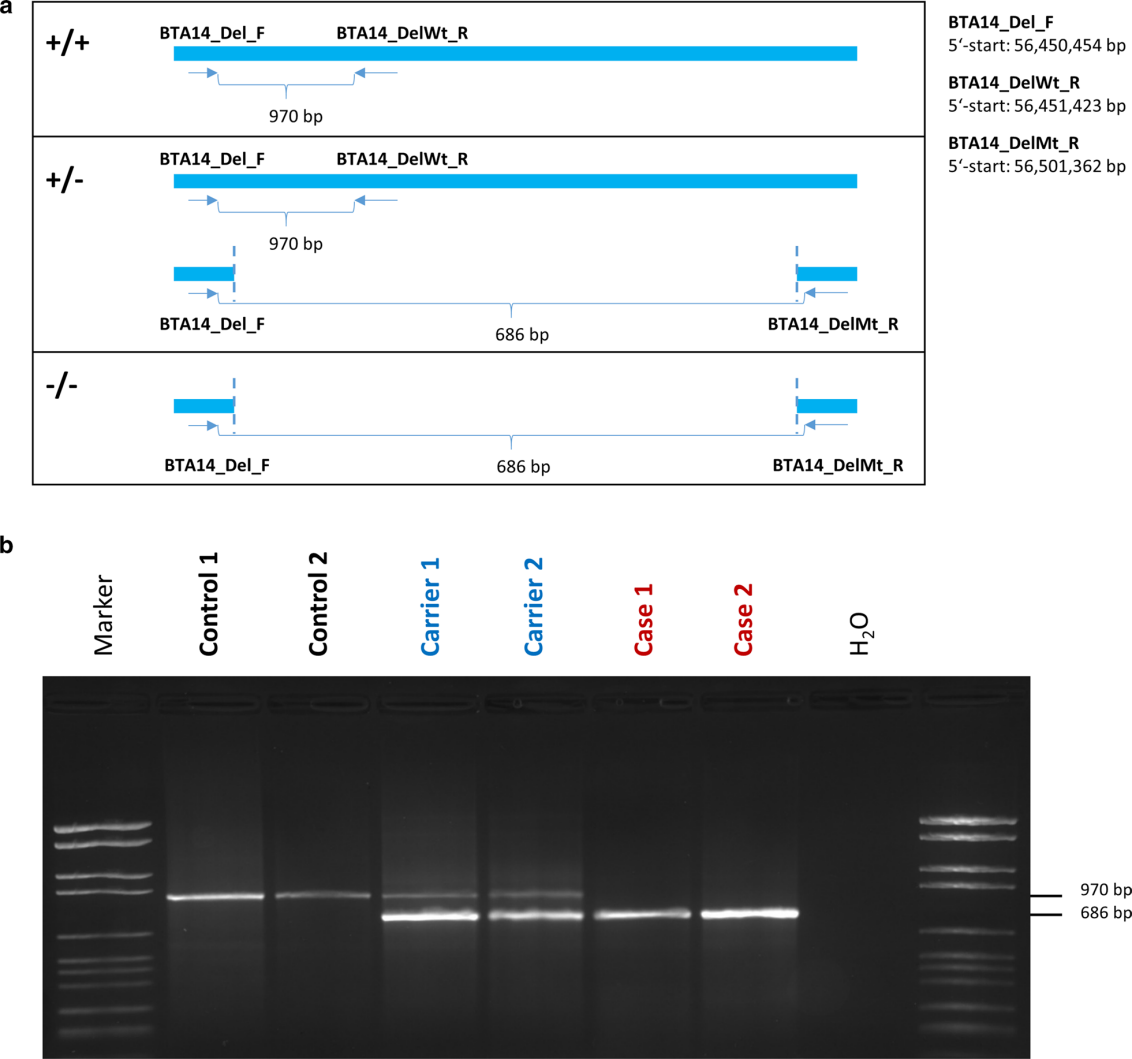
PCR amplification of structural variant. **a** Schematic drawing of the location of PCR primers. Two PCR primer pairs using the same forward primer (BTA14_Del_F) were designed. PCR amplification for homozygous wildtype (+/+), heterozygous (+/−) and homozygous mutated (−/−) state is shown. The forward primer is located upstream the deletion boundary. Primer BTA14_DelWt_R is located in the deleted region. Amplified PCR product size is predicted to be 970 bp long. Another reverse primer is located downstream of the deletion boundary. The 686-bp PCR product is obtained only if the region between forward and reverse primer is deleted. **b** Agarose gel picture of the two PCR products for two controls, two carriers and two cases

PCR on genomic DNA was used to confirm the co-segregation of the deletion with the disease phenotype in all available family members (n = 23) and to identify the exact deletion breakpoints. The use of two primer combinations resulted in two distinct products (F and DelWt_R, F and DelMt_R, see Fig. 4a): a longer 970-bp fragment representing the wild type allele, and a shorter 686-bp fragment, specific to the deletion allele (Fig. 4b). In addition to the sire, three dams and six offspring were heterozygous for the deletion. Only the malformed animals were homozygous for the deletion. Furthermore, the deletion was not present in 182 unrelated Holstein Friesians. Sanger sequencing of PCR products of two homozygous cases and two heterozygous carriers revealed that the deletion breakpoints are at positions 56,451,029 bp and 56,501,201 bp on BTA14. Moreover, six additional base pairs inserted into the deleted region were detected. Eventually, the variant was defined with respect to the ARS-UCD1.2 assembly as BTA14 g.56,451,029–56,501,201delinsTGACAA.

**Fig. 5:**
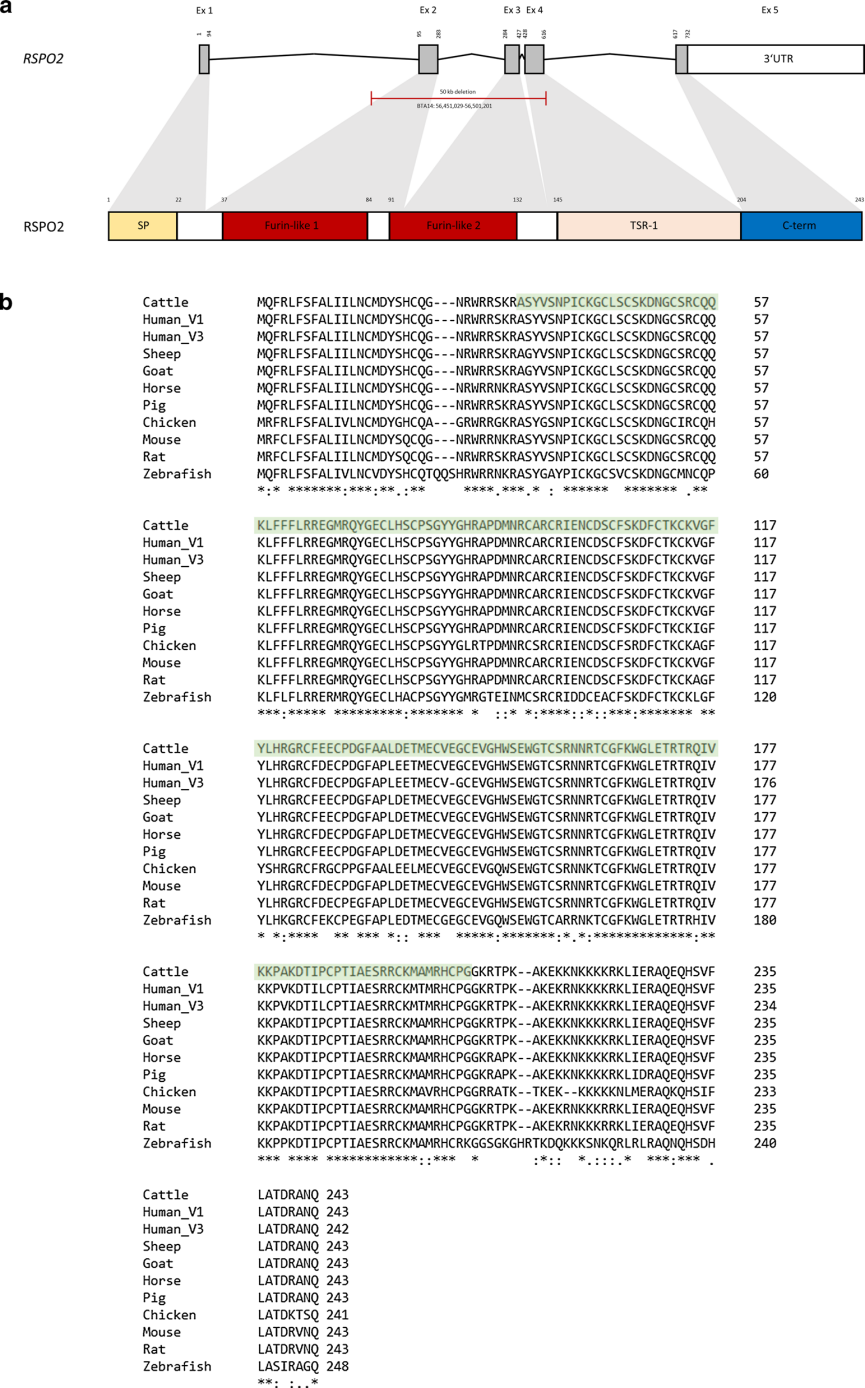
Genomic and protein structure of bovine *RSPO2*. **a** *RSPO2* genomic (top) and protein (bottom) structures with identified structural variant. Protein domains are indicated. Ex, exon. UTR, untranslated region. Adapted from Szenker-Ravi et al. [22] **b** Multispecies alignment of the RSPO2 protein sequence. The amino acids affected by the deletion are indicated in light green; RSPO2 is highly conserved across investigated vertebrate species. The sequences for the alignment were taken from the following accessions: NP_001193021.1 (cattle), NP_848660.3 and XP_016868884.1 (human), XP_014953325.1 (sheep), XP_005689216.1 (goat), NP_001103151.1 (horse), NP_001280070.1 (pig), NP_001305953.1 (chicken), NP_001344885.1 (mouse), XP_008763677.1 (rat) and NP_001268919.1 (zebrafish)

According to the annotation of the bovine genome (ARS-UCD.1.2, National Center for Biotechnology Information (NCBI) release 106), the deleted sequence contains three exons of the *R-spondin 2* gene (*RSPO2*; NM_01206092.2) (Fig. 5a). In silico analysis showed that these exons encode three protein domains and 174 amino acids, which account for 71.6% of the entire protein. Multispecies alignment of RSPO2 revealed that the protein sequence is highly conserved across all investigated vertebrate species (Fig. 5b).

## Discussion

Our findings provide evidence that supports the association between homozygosity of a 50-kb deletion on BTA14 and tetradysmelia in Holstein Friesian cattle. The use of homozygosity mapping in combination with whole-genome sequencing enabled us to identify a candidate region and a plausible causal structural variant. In affected animals, the mutation might have occurred as a de novo variant in the sire. In contrast to the affected calves’ sire, the grandsire had several thousand offspring, and was a bull sire in conventional breeding programs in the 1980s worldwide, but no other affected offspring have been reported. Due to the fact that (i) we did not have access to ancestral DNA samples to test whether the 50-kb deletion was already present in the sire’s maternal or paternal lineage, (ii) tetradysmelia is a rare genetic disorder [11], and (iii) there is no comprehensive reporting system for congenital malformations in cattle across Germany, we cannot confirm if the 50-kb deletion is a de novo mutation in the sire of the affected calves. Furthermore, we hypothesized that, within the 1000 Bulls Genome dataset, the causal variant is exclusively present in the heterozygous state in the sire since tetradysmelia is a rare disorder in Holsteins and has not been reported in other cattle breeds, to date. However, this approach might have limited the identification of rare variants that are present not only in the sire but also in other Holsteins of the 1000 Bulls Genome dataset.

As a result of the backcrossing, non-affected family members shared some of the other homozygous genome segments that were identified by homozygosity mapping and had alleles identical to those of the malformed individuals, which helped us to focus on the 10.54-Mb chromosome segment on BTA14. Mapping of older mutations might have required larger numbers of affected animals, since the associated IBD haplotype is usually much smaller due to independent recombination events across several generations [34].

In humans, limb malformations are not rare and occur with a frequency of approximately one in a thousand births [35]. Unfortunately, the true frequency of limb malformations in cattle is unknown and not well recorded due to lack of surveillance programs. However, there are several scientific reports about bovine limb malformations from as early as the 1920s (Table 1) [2, 36–40]. Those studies have reported that affected calves had limb defects distal of the knee and elbow. In addition, calves with akroteriasis congenita and “amputated” calves show severe craniofacial deformities, e.g. mandibular hypoplasia, hydrocephalus and/or cleft lip/palate [36–38]. Causal mutations for these disorders in cattle are still unknown and non-genetic factors are discussed. For instance in humans, disruptive events during the gestation, such as amniotic band or vascular disruptions, may cause amputation or hypoperfusion of developing limbs, and result in malformations [41]. Furthermore, various congenital limb defects may be caused by prenatal exposure to various teratogens [42]. The best-known example of a pharmaceutical teratogen is thalidomide, which caused a wide range of limb malformations in humans in the 1960s, especially intercalary reductions and preaxial defects [43]. Nevertheless, from the analysis of the pedigree of the backcrossed Holstein Friesian family, of the malformation phenotype, and of the sex ratio of affected animals, we conclude that tetradysmelia in this family is a genetic disorder inherited in an autosomal recessive mode. In addition, the malformed animals were born on different farms, which reduces the possibility of a non-genetic factor. The same is true for possible causal effects of superovulation and embryo transfer. In the literature, there are no reports about limb malformations associated with these procedures in spite of more than 750,000 bovine embryos produced annually worldwide [44].

Previous homozygosity mapping studies with microsatellite markers and candidate gene analyses excluded genes, which are known to cause similar phenotypes in mice, as causal loci for tetradysmelia in cattle [23–26, 45]. To date, no congenital defect, identical to the tetradysmelia that we describe in our study, has been reported in other cattle breeds, although some historical reports on severe limb malformations exist (Table 1) [36–40]. However, in humans a similar condition exists, i.e., tetra-amelia, which is an extremely rare event (4/10 million births; [46]), with foetuses lacking all four limbs completely. This disorder is often linked with other defects, e.g. lung hypoplasia, heart wall defects, cleft lip/palate, urogenital and craniofacial defects [22, 47–50]. Necropsy of bovine tetradysmelia cases did not show any gross malformations of the lungs or other internal organs.

An absence of all limbs was also described in chickens [13]. Similar to the phenotype of affected cattle, the pectoral and pelvic girdles were fully formed in birds. In addition, limbless chickens had a smaller upper beak. Two of the tetradysmelia cattle individuals also had craniofacial deformities: one had a shortened upper jaw (brachygnathia superior) and one had the mandibulae with an incomplete symphyseal fusion. However, it is not clear if these malformations are associated with the identified tetradysmelia genetic defect or just incidental findings.

Interestingly, tetra-amelia seems to be compatible with life [51], whereas the tetradysmelic animals were stillborn. One reason for this difference might be that dystocia as a result of missing limbs, which are important for successful initiation of birth in cattle [52], occurs only in cattle and not in humans. Alternatively, there may be a physiological incompetence of the tetradysmelic animals to shift from intra- to extrauterine life.

In 2004, Niemann et al. [20] reported a mutation in *WNT3* that causes tetra-amelia in humans, and in 2011, Eyaid et al. [21] discovered a mutation in *WNT7A* that causes a very similar phenotype in humans. Wnt signalling is involved in limb development and controls dorsal/ventral patterning [19]. It also regulates bone resorption and formation of new bone [53, 54]. Only recently, two mutations in *RSPO2* have been identified in human tetra-amelia [22]. The protein R-spondin 2 encoded by *RSPO2* is a secreted ligand that enhances Wnt signalling [55] and is expressed, inter alia, in the limb buds [56, 57]. R-spondin 2 binds to its cognate receptors via its furin-like 2 domain and to ubiquitin ligases via its furin-like 1 domain [58–60], thereby preventing Wnt receptor degradation. The identified 50-kb deletion in cattle presumably affects both domains since the exons that are deleted in *RSPO2* and encode those domains are located within the affected genomic region. Different types of mutations were identified by Szenker-Ravi et al. [22] that ranged from a point mutation in the upstream region of the human *RSPO2* gene, to nonsense and frameshift mutations, and to a deletion of the last intron and exon. Notably, all malformed foetuses in the study of Szenker-Ravi et al. [22] not only showed severe limb malformations or lack of all limbs, but also displayed other various phenotypes that correlated with the degree to which RSPO2 signalling was impaired by the mutations. Mice that lack *Rspo2* die shortly after birth and display anomalies related to limb and craniofacial skeleton development, and to lung and kidney development [61]. Interestingly, *rspo2*^*null*^ zebrafish mutants do not form fin ray skeletons and show hypoplasia of the neural/haemal arches and ribs [62]. Therefore, the role of *RSPO2* in limb development seems to be highly conserved in mammalian and non-mammalian vertebrate species.

## Conclusions

In summary, we identified a 50-kb deletion on BTA14 in a Holstein Friesian family, which most likely disrupts the bovine *RSPO2* gene. Consequently, the Wnt signalling pathway may be impaired and limb development disrupted. Therefore, cattle that are homozygous for this deletion presumably suffer from tetradysmelia. To our knowledge, this is the first reported candidate causal mutation for tetradysmelia in a large animal model. Since signalling systems in limb development are conserved across species, the observed inherited defect may serve as a model to further elucidate fundamental pathways involved in limb development.

## Supplementary information

**Additional file 1:Table S1.** Candidate genes involved in limb development and formation. Candidate genes used for variant filtering of whole-genome sequencing data and involved in limb development and formation were chosen based on their reported association with limb malformations in other species as well as the knowledge about the gene's biological function. References were taken from the Online Mendelian Inheritance in Animals catalogue (OMIA) and the Online Mendelian Inheritance in Man (OMIM) records.

**Additional file 2: Figure S1** Whole-genome sequencing analysis in Integrative Genomics Viewer (IGV). IGV screen shot of the region on BTA14 (NC_037341.1) from 56,444,864 bp to 56,512,976 bp. Mapped reads are represented by grey bars. Note that the coverage drops between 56.45 and 56.50 Mb. The red bars represent paired reads that have an average insert size of 50,100 bp.

## Data Availability

Genotypes and whole-genome sequence data are available from the authors upon reasonable request.
