# Supplementary material for: A 50-kb deletion disrupting the RSPO2 gene is associated with tetradysmelia in Holstein Friesian cattle
Source: Genet Sel Evol. 2020 Nov 11;52:68. doi: 10.1186/s12711-020-00586-y (PMC7661195; doi:10.1186/s12711-020-00586-y)
Supplement: Supplementary file 1 — Additional file 1:Table S1. Candidate genes involved in limb development and formation. Candidate genes used for variant filtering of whole-genome sequencing data and involved in limb development and formation were chosen based on their reported association with limb malformations in other species as well as the knowledge about the gene's biological function. References were taken from the Online Mendelian Inheritance in Animals catalogue (OMIA) and the Online Mendelian Inheritance in Man (OMIM) records. [file 12711_2020_586_MOESM1_ESM.pdf]

Table S1. Genes involved in limb development and formation

Gene	Name	BTA (ARS-UCD1.2)	Reference(s)
IHH	indian hedgehog signaling molecule	2	https://omia.org/OMIA000006/9031/; https://omim.org/entry/112500; https://omim.org/entry/607778
NPPC	natriuretic peptide C	2	https://omim.org/entry/600296
SHOX	short stature homeobox	2	https://omia.org/OMIA002013/9796/; https://omim.org/entry/312865
ITGA10	integrin subunit alpha 10	3	https://omia.org/OMIA001886/9615/
COL9A2	collagen type IX alpha 2 chain	3	https://omia.org/OMIA001523/9615/; https://omim.org/entry/614284
COL11A1	collagen type XI alpha 1 chain	3	https://omim.org/entry/120280
DDR2	discoidin domain receptor tyrosine kinase 2	3	https://omim.org/entry/191311
SLC13A1	solute carrier family 13 member 1	4	https://omia.org/OMIA001400/9940/; https://omia.org/OMIA001315/9615/
COL2A1	collagen type II alpha 1 chain	5	https://omia.org/OMIA001926/9823/; https://omia.org/OMIA001926/9913/; https://omim.org/entry/200610
SUOX	sulfite oxidase	5	https://omia.org/OMIA000059/9913/
EVC2	EvC ciliary complex subunit 2	6	https://omia.org/OMIA000187/9913/; https://omim.org/entry/225500
FGFR3	fibroblast growth factor receptor3	6	https://omia.org/OMIA001703/9913/; https://omia.org/OMIA001703/9940/; https://omim.org/entry/134934
COMP	cartilage oligomeric matrix protein	7	https://omim.org/entry/600310
SLC26A2	solute carrier family 26 member 2	7	https://omim.org/entry/606718
HAPLN1	hyaluronan and proteoglycan link protein 1	7	https://omim.org/entry/115435
NPR2	natriuretic peptide receptor 2	8	https://omim.org/entry/108961
COL10A1	collagen type X alpha 1 chain	9	https://omia.org/OMIA001718/9823/; https://omim.org/entry/120110
COL9A1	collagen type IX alpha 1 chain	9	https://omim.org/entry/120210
BMP4	bone morphogenetic protein 4	10	https://omim.org/entry/112262
GREM1	gremlin 1, DAN family BMP antagonist	10	https://omia.org/OMIA001061/9925/; https://omim.org/entry/603054
FMN1	formin 1	10	https://omia.org/OMIA001061/9925/; https://omim.org/entry/136535
MATN3	matrilin 3	11	https://omim.org/entry/602109
GDF5	growth differentiation factor 5	13	https://omim.org/entry/601146
COL9A3	collagen type IX alpha 3 chain	20	https://omia.org/OMIA001522/9615/; https://omim.org/entry/120140
ACAN	aggrecan	21	https://omia.org/OMIA001271/9796/; https://omia.org/OMIA001271/9913/; https://omia.org/OMIA000702/9031/; https://omim.org/entry/155760
COL11A2	collagen type XI alpha 2 chain	23	https://omia.org/OMIA001772/9615/; https://omim.org/entry/120290
MOCS1	molybdenum cofactor synthesis 1	23	https://omia.org/OMIA001541/9913/
FGF4	fibroblast growth factor 4	29	https://omia.org/OMIA002133/9615/; https://omim.org/entry/164980
