# Supplementary figures and images for: A 50-kb deletion disrupting the RSPO2 gene is associated with tetradysmelia in Holstein Friesian cattle
Source: Genet Sel Evol. 2020 Nov 11;52:68. doi: 10.1186/s12711-020-00586-y (PMC7661195; doi:10.1186/s12711-020-00586-y)

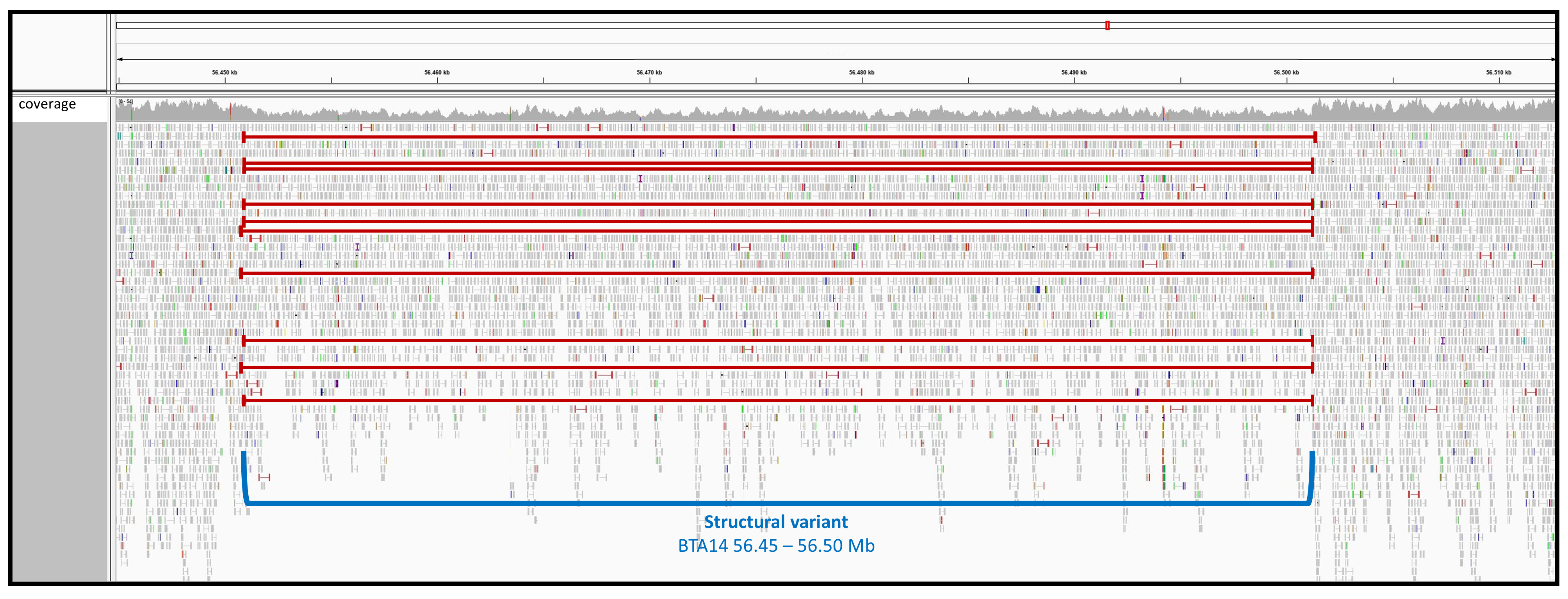

Supplement: Supplementary file 2 — Additional file 2: Figure S1 Whole-genome sequencing analysis in Integrative Genomics Viewer (IGV). IGV screen shot of the region on BTA14 (NC_037341.1) from 56,444,864 bp to 56,512,976 bp. Mapped reads are represented by grey bars. Note that the coverage drops between 56.45 and 56.50 Mb. The red bars represent paired reads that have an average insert size of 50,100 bp. [file 12711_2020_586_MOESM2_ESM.tif]
